# Rapid detection of residual chlorpyrifos and pyrimethanil on fruit surface by surface-enhanced Raman spectroscopy integrated with deep learning approach

**DOI:** 10.1038/s41598-023-45954-y

**Published:** 2023-11-13

**Authors:** Zhu Chen, Xuan Dong, Chao Liu, Shenghao Wang, Shanshan Dong, Qing Huang

**Affiliations:** 1 Science Island Branch of Graduate School, University of Science and Technology of China, Hefei, China; 2 Anhui Province Key Laboratory of Aquaculture and Stock Enhancement, Fisheries Research Institution, Anhui Academy of Agricultural Sciences, Hefei, China; 3 CAS Key Laboratory of High Magnetic Field and Ion Beam Physical Biology, Anhui Key Laboratory of Environmental Toxicology and Pollution Control Technology, Institute of Intelligent Machines, Hefei Institute of Intelligent Agriculture, Hefei Institutes of Physical Science, Chinese Academy of Sciences, Hefei, China; 4 Department of Basic Sciences, Army Academy of Artillery and Air Defense, Hefei, China; 5 Henan Key Laboratory of Ion-Beam Bioengineering, School of Physics and Microelectronics, Zhengzhou University, Zhengzhou, China

**Keywords:** Analytical chemistry, Statistics

## Abstract

Chlorpyrifos and pyrimethanil are widely used insecticides/fungicides in agriculture. The residual pesticides/fungicides remaining in fruits and vegetables may do harm to human health if they are taken without notice by the customers. Therefore, it is important to develop methods and tools for the rapid detection of pesticides/fungicides in fruits and vegetables, which are highly demanded in the current markets. Surface-enhanced Raman spectroscopy (SERS) can achieve trace chemical detection, while it is still a challenge to apply SERS for the detection and identification of mixed pesticides/fungicides. In this work, we tried to combine SERS technique and deep learning spectral analysis for the determination of mixed chlorpyrifos and pyrimethanil on the surface of fruits including apples and strawberries. Especially, the multi-channel convolutional neural networks-gate recurrent unit (MC-CNN-GRU) classification model was used to extract sequence and spatial information in the spectra, so that the accuracy of the optimized classification model could reach 99% even when the mixture ratio of pesticide/fungicide varied considerably. This work therefore demonstrates an effective application of using SERS combined deep learning approach in the rapid detection and identification of different mixed pesticides in agricultural products.

## Introduction

In order to prevent diseases and invasion of pests to the plants, pesticides and fungicides are widely applied in agriculture, usually sprayed on the surface of fruits and vegetables. Chlorpyrifos and pyrimethanil are widely used insecticides/fungicides in the world and have been shown to be effective in controlling fruit and vegetable pests and gray mold^1^. Chlorpyrifos is a best-selling organophosphate insecticide, while its accumulation in the body may cause a variety of diseases and even affect the neurological development of children^2^. Pyrimethanil is a bactericidal agent widely used in agriculture. It belongs to aniline-pyrimethanil bactericides, which can prevent the infection of bacteria and kill bacteria by inhibiting the production of pathogen disseminated enzymes^3^. The combination application of multiple pesticides and fungicides is very common in agricultural production^4^. However, inappropriate application of pesticides and fungicides may pose a serious threat to human health. If they are not noticed and taken carelessly serious health problems may occur^5^. Therefore, it is very important to develop simple, accurate and convenient detection methods, especially in the case of use of multiple pesticides/fungicides^6^.

Currently, many methods have been developed for the detection of pesticide/fungicide residues in food, such as liquid chromatography–mass spectrometry (LC–MS/MS)^7,8^, gas chromatography–mass spectrometry (GC–MS)^9^, etc. Although some of these methods have become national standard methods in examination of pesticides/fungicides, they also show limitation in the application due to their complex operation, special working environment and special sample storage conditions^10^.

Raman spectroscopy has attracted much attention due to its nondestructive analytical ability, and it can provide rich information about the chemical components, structures, molecular vibrations and interactions of the measured samples^11^. Especially, due to the development of surface-enhanced Raman spectroscopy (SERS), Raman spectroscopy is now widely used in many fields^12–15^. Actually, SERS has been employed to detect trace pesticide residues in foods^16,17^. Recently, a new trend of using SERS technique combined with stoichiometry and deep learning approaches has emerged, which shows improved performance in identifying mixed pesticides^18^. Especially, compared with other deep learning models, the Convolutional Neural Network (CNN) model, due to its characteristics of sparse connections and weight sharing for reducing the risk of overfitting^19^, shows the special potential in the application of Raman spectral analysis as it requires few or no spectral data preprocessing steps^20^.

Therefore, in this work we attempted to explore the CNN-based deep-learning model for the SERS detection and identification of mixed pesticides/fungicides in fruits. For this purpose, we employed SERS to measure chlorpyrifos, pyrimethanil and mixed pesticide/fungicide residues on the surface of apples and strawberries, and applied a multi-channel convolutional neural networks-gate recurrent unit (MC-CNN-GRU) classification model to achieve a semi-quantitative analysis of mixed pesticide/fungicide residues on the fruit samples. The results showed improved accuracy in the determination of the mixed pesticide/fungicide on the fruit surface.

## Materials and methods

### Reagents and fruit samples

Chlorpyrifos (99.9%) and pyrimethanil (99.1%) solids were purchased from Tanmo Quality Inspection Technology Co., Ltd. Analytical purity acetonitrile (≥ 99.7%) and 4-Aminothiophenol (98%) were obtained from Bioengineering Co., China. For the preparation of gold nanoparticles (AuNPs) for SERS detection, chloroauric acid(≥ 99.9%) and trisodium citrate (≥ 99.5%) were purchased from Sinopharm Chemical Reagent Co., Ltd. Samples of strawberries and apples were randomly purchased in the markets.

### Fabrication and characterization of AuNPs

Au nanoparticles were prepared by Frens method reported in the literature^21^, and some adjustments were made. Briefly, 120 mL ultrapure water was put into a conical flask and heated to boiling. Then 7 mL 1% C_6_H_5_Na_3_O_7_ solution and 1 mL 1% HAuCl_4_ solution were added successively and heated for 20 min. The prepared gold nanoparticles were stored in the refrigerator at 4 °C. The UV–Vis spectrum was measured by Shimazu UV-2550 spectrophotometer, and the TEM results were obtained by Escalab 250Xi transmission electron microscope. A series of 4-Aminothiophenol (4-ATP) probe standard solutions were used to verify the activity of AuNPs.

### SERS measurements

Apple and strawberry samples were bought from markets. Chlorpyrifos and pyrimethanil mixed solutions were prepared by mixing them at different concentrations according to the volume ratio (1:1, 1:2, 1:3, 1:4, 4:1, 3:1, 2:1, 1:1 chlorpyrifos/pyrimethanil). 600 μL standard pesticide/fungicide solution (with different concentrations and ratios) was dropped on the 1 cm × 1 cm area of the fruit surface. After the solution was dried at room temperature, the surface of the fruit surface was wiped with a wetted cotton swab. Then the swab was put into a centrifuge tube containing 0.5 mL acetonitrile. Then, after 60 W ultrasound for 5 min, the obtained liquid was ready for SERS detection.

For the SERS detection, 100 μL liquid was first taken into a quartz dish, then 500 μL AuNPs was added. Then, 100 μL 5% NaCl solution was added. Raman spectra were obtained using Ocean Optics RMS 1000 portable Raman spectrometer (785 nm). Each measurement was repeated at least three times.

### Density functional theory (DFT) calculation

The DFT calculation of chlorpyrifos and pyrimethanil was calculated by Gaussian 09 software. The calculations in this work were performed by applying the functionals M06-2X and 6-311 + G (d,p) basis set. The tight convergence criterion of Gaussian was adopted for structural optimization, and the ultrafine integration grid was used in the numerical integration of the structure optimization and vibrational frequencies calculation. The geometries were fully optimized without any constraint on the geometry and the optimized structures had no imaginary frequencies. The calculation of harmonic vibrational wavenumbers and relative Raman activity was carried out at the same theoretical level using the same basis set. The calculated Raman activity was converted to Raman intensity by Multiwfn wavefunction analysis program using the relation derived from the basic theory of Raman scattering.

### Data analysis

Before the data analysis, normalization and Savizkg–Golag (SG) smoothing data treatment was applied to the SERS spectra. In addition, the second-order polynomial fit was used for baseline correction to eliminate the effect of the fluorescent background. The peak intensity was evaluated using mean value ± standard deviation.

For the establishment of quantification or qualification models, before we tried the deep learning approach, we first tried the method of Principal Component Analysis (PCA), which converts a group of linearly correlated variables into a group of nonlinearly correlated variables through orthogonal transformation^22,23^. In this process, the important features related to the target observation in the high-dimensional data are retained, and the noise and irrelevant features are removed. Principal component analysis transforms the original N-dimensional data space into K-dimensional orthogonal coordinate space with a large variance contribution rate. Besides, we also applied support vector machines (SVM), which is to find the plane with the maximum interval that can correctly partition the data set^24^. For linearly indivisible data sets, SVM can transform the known data space into a high-dimensional space through the nonlinear mapping of the kernel function, and replace the original nonlinear problem with the transformed linear problem for solutions. Finally, to improve the prediction accuracy, we tried CNN-GRU model which is a hybrid prediction model combining Convolutional Neural Network (CNN) and Gated Recurrent Unit (GRU)^25^. Feature extraction in CNN is realized through the dot product operation between the translation of the convolution kernel on the original data and the corresponding receptor field^26^. The sequence correlation of information is ignored in the process. GRU can process sequence information in spectra. Compared with Long Short-Term Memory (LSTM), which requires multiple gating, GRU only needs one gating to complete the forgetting and selective memory of data information, which saves time for the training of big data samples^27^. Therefore, MC-CNN-GRU takes multiple channels to input data, which can learn more complete feature information and eliminate errors caused by uneven samples. The MC-CNN-GRU and SVM qualitative model was established using the program provided by Matlab 2022a.

### Ethical approval

We confirmed that all plant experiments were conducted in accordance with relevant institutional, national, and international guidelines and legislation.

## Results and discussion

### Characterization of AuNPs

**Figure 1:**
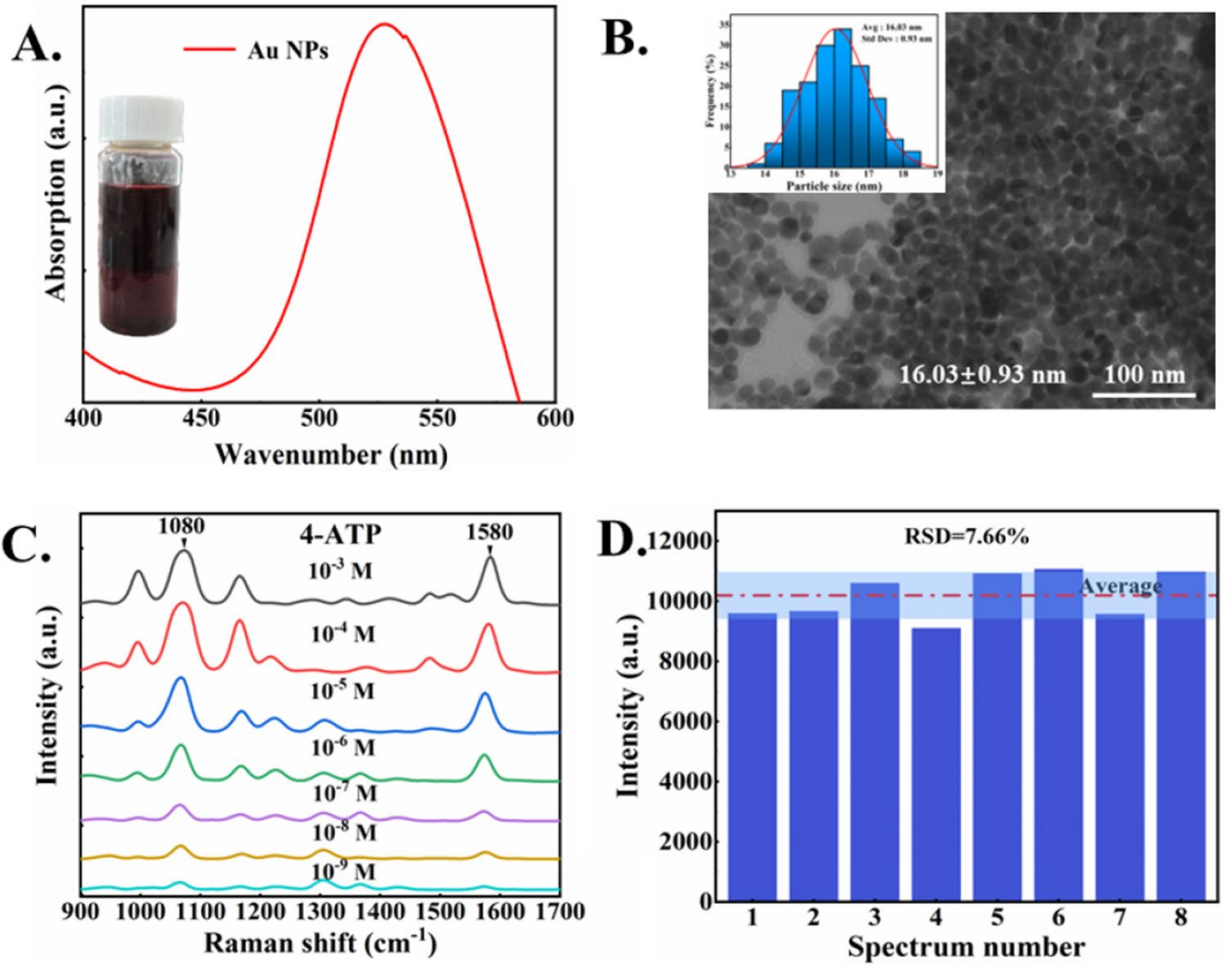
(**A**) UV–Vis spectrum of AuNPs. (**B**) TEM result of AuNPs. (**C**) SERS spectra of 4-ATP probes with different concentrations. (**D**) Characteristic peak intensity of SERS spectra at 1580 cm^−1^ with the same concentration of 4-ATP.

The UV–Vis spectrum of the synthesized AuNPs in Fig. 1A shows a sharp absorption peak, which indicates the successful synthesis of AuNPs with the relatively uniform shape and size of the nanoparticles. The peak at 528 nm belongs to the plasmon resonance band^28^. The TEM image in Fig. 1B confirms the uniform spherical structures of AuNPs, with the averaged diameter of (16.03 ± 0.93) nm.

In order to verify the activity of AuNPs, the 4-ATP (4-Aminothiophenol) was used as the standard probe. Figure 1C shows the SERS spectra of 4-ATP with the characteristic peaks at 1080 cm^−1^ and 1580 cm^−1^^29^, indicating the lower limit of detection (LOD) reaching as low as 10^−9^ M. In the SERS detection, the reproducibility of the substrate is crucial to the stability of the detection results. Figure 1D presents the SERS spectra from 8 different samples, showing no significant difference in the intensity for these samples, with the relative standard deviation (RSD) is about 7.66%, confirming the reproducibility of the SERS measurements based on the as-prepared AuNPs.

### Spectral characteristic peaks of the pesticide/fungicide

**Figure 2:**
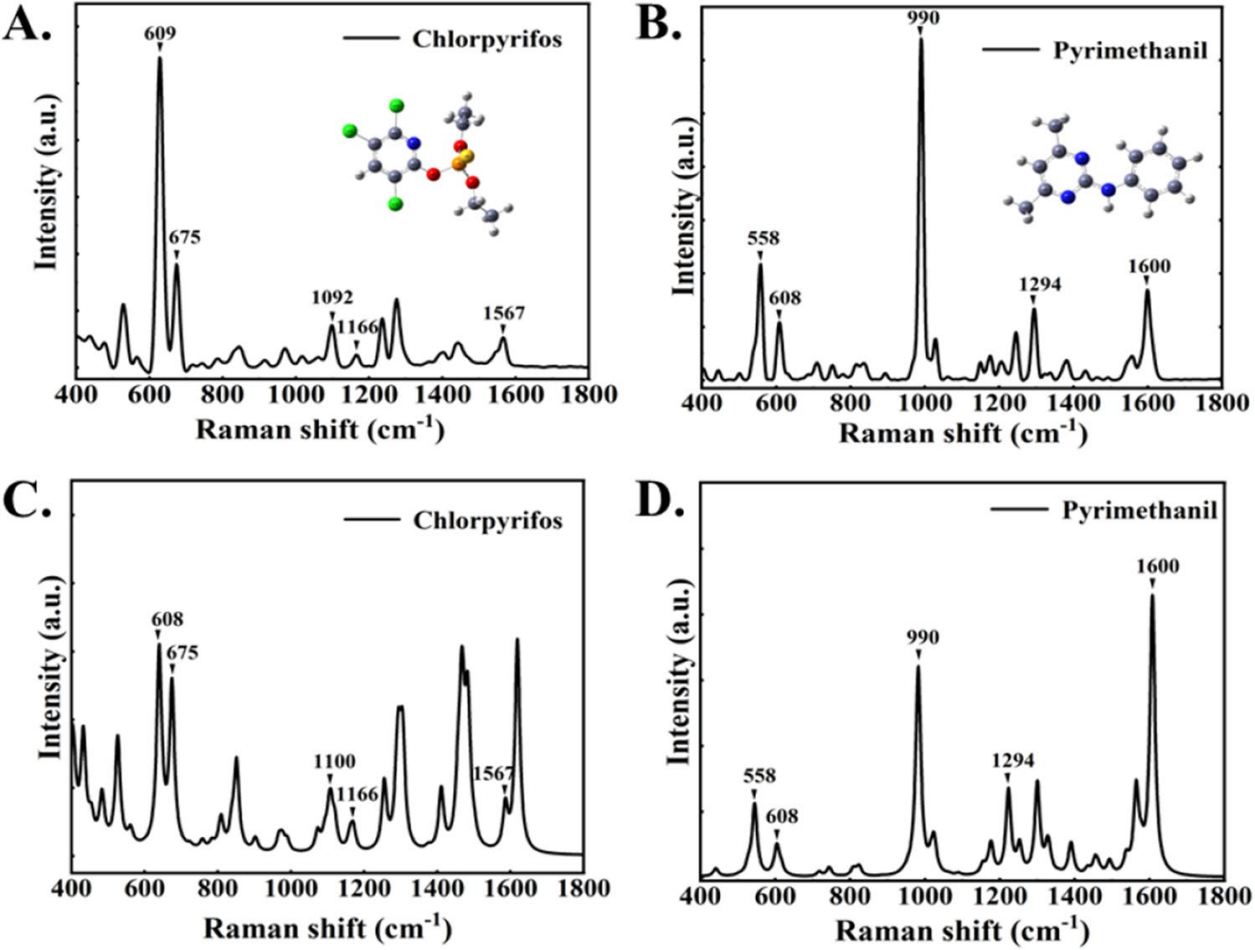
(**A**) Molecular structure and Raman spectra of chlorpyrifos and (**B**) pyrimethanil solid powders. (**C**) Chlorpyrifos and (**D**) pyrimethanil DFT calculation spectra.

The Raman spectra of chlorpyrifos and pyrimethanil are shown in Fig. 2A,B, and the DFT calculated spectra of chlorpyrifos and pyrimethanil are shown in Fig. 2C,D, respectively. Comparative analysis shows that the Raman spectra and DFT calculation spectra of the both chlorpyrifos and pyrimethanil are consistent.

The main Raman characteristic peaks of chlorpyrifos solid powder are 609 cm^−1^, 668 cm^−1^ and 1092 cm^−1^, and the main Raman characteristic peaks of pyrimethanil solid are 559 cm^−1^, 608 cm^−1^, 990 cm^−1^, 1294 cm^−1^ and 1600 cm^−1^. To be noted, the relative intensity of some bands in the SERS spectrum may change with concentration. This is because SERS is a very sensitive tool for detection of the molecules adsorbed on the surface of the nanoparticles, or trapped in the gap of the nanoparticles (so called “hotspots”). So there could be possible interactions between the analytes and the SERS nanoparticles, resulting in the uncertain intensity. Nevertheless, the position of each peak in the Raman spectrum can be identified and determined. Raman spectra of chlorpyrifos and pyrimethanil solution without gold nanoparticles are compared with the SERS spectra of 10^−3^ M standard solution. The peaks at 740 cm^−1^, 910 cm^−1^, 1040 cm^−1^ and 1370 cm^−1^ are solvent peaks, which do not overlap with the characteristic peaks of chlorpyrifos and pyrimethanil.

### Analysis of SERS spectra of standard solutions

**Figure 3:**
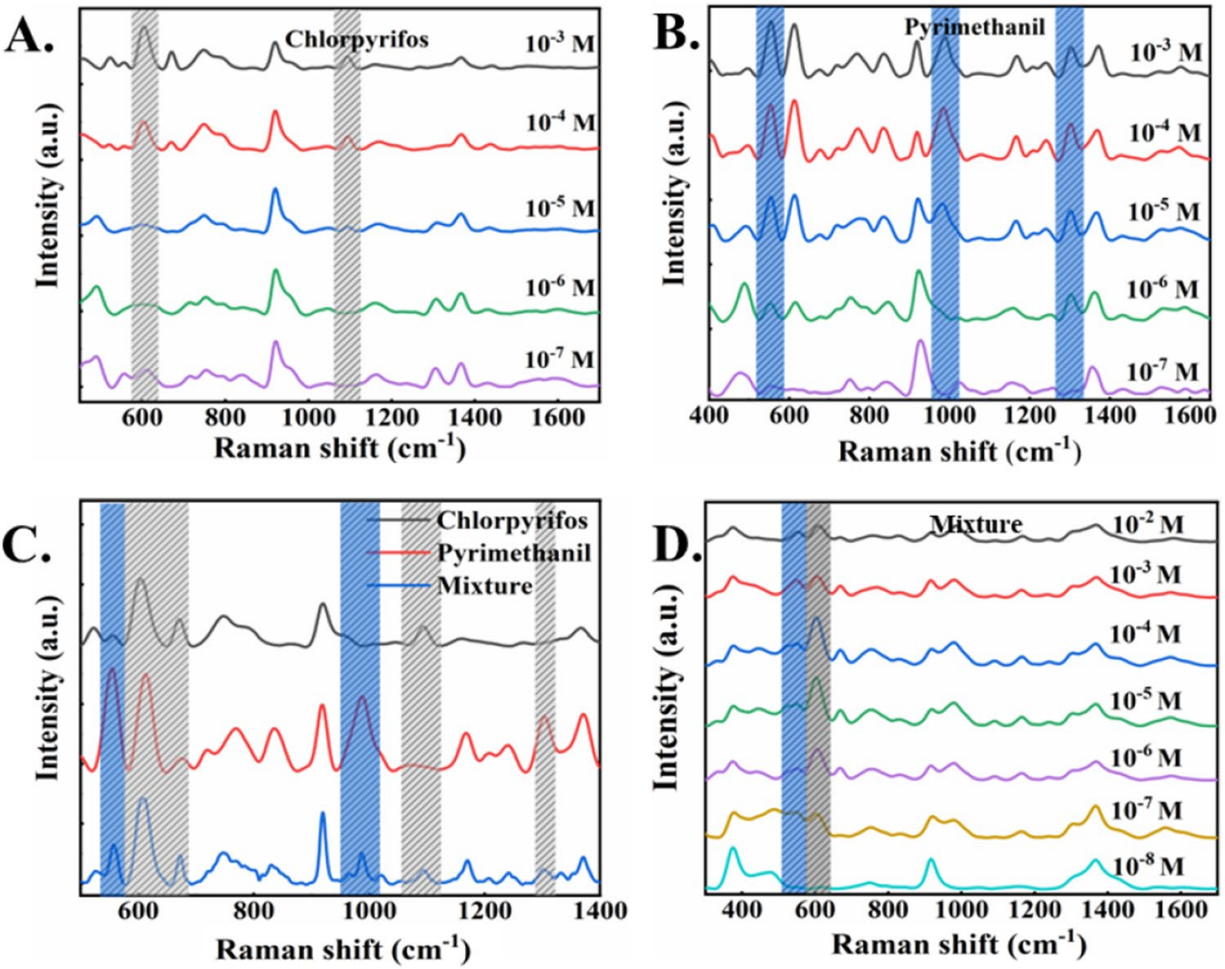
(**A**) SERS spectra of chlorpyrifos with different concentrations. (**B**) SERS spectra of pyrimethanil with different concentrations. (**C**) SERS spectra of chlorpyrifos, pyrimethanil and mixture (equal volume) with the same concentration. (**D**) SERS spectra of the mixture (equal volume) with different concentrations.

SERS detection was conducted for different concentrations of chlorpyrifos, pyrimethanil and their mixtures, and the results are shown in Fig. 3. In the SERS spectra of the standard chlorpyrifos solutions (Fig. 3A), corresponding characteristic peak can be clearly observed at 609 cm^−1^, 668 cm^−1^ and 1092 cm^−1^. In Fig. 3B, the characteristic peaks of pyrimethanil at 559 cm^−1^, 990 cm^−1^, 1294 cm^−1^ and 1600 cm^−1^ are also clearly identified. Detailed Raman band assignments are given in Table 1.

**Table 1:** Attribution of SERS characteristic peaks of chlorpyrifos and pyrimethanil.

Analyte	SERS/cm^−1^	Assignment
Chlorpyrifos^30^	609	P=S
	675	P=S
	1092	P–O–C stretch
	1166	Cl-ring, δ(C_H)
	1567	Ring stretching
Pyrimethanil^31^	558	Antisymmetric stretching
	608	Aromatic ring in plane deformation
	990	Breathing mode aromatic ring
	1294	Antisymmetric stretching
	1600	CH_ring_ Antisymmetric stretching

A comparative analysis of Raman spectra of chlorpyrifos and pyrimethanil solid powder and SERS spectra of their standard solutions shows that their characteristic peaks have slight Raman frequency shift in SERS detection. Figure 3C shows the SERS spectra of the standard solution of chlorpyrifos, pyrimethanil and their mixtures. By comparing their SERS spectra, it can be found that the characteristic peaks of chlorpyrifos and pyrimethanil in the SERS spectra of the mixed solution changed in intensity with concentration. This proves the feasibility of surface-enhanced Raman spectroscopy in the detection of mixed pesticides/fungicides. Figure 3D shows SERS spectra of equal volume mixture with different concentrations. The Raman characteristic peaks of chlorpyrifos and pyrimethanil are clearly observed in the mixed samples. The SERS characteristic peaks at 609 cm^−1^ in chlorpyrifos and 558 cm^−1^ in pyrimethanil with low concentration (ca. 10^−7^ M) can be identified clearly, confirming the high sensitivity of the SERS measurements of both chlorpyrifos and pyrimethanil.

### Analysis of SERS spectra of chlorpyrifos and pyrimethanil on fruit surface

**Figure 4:**
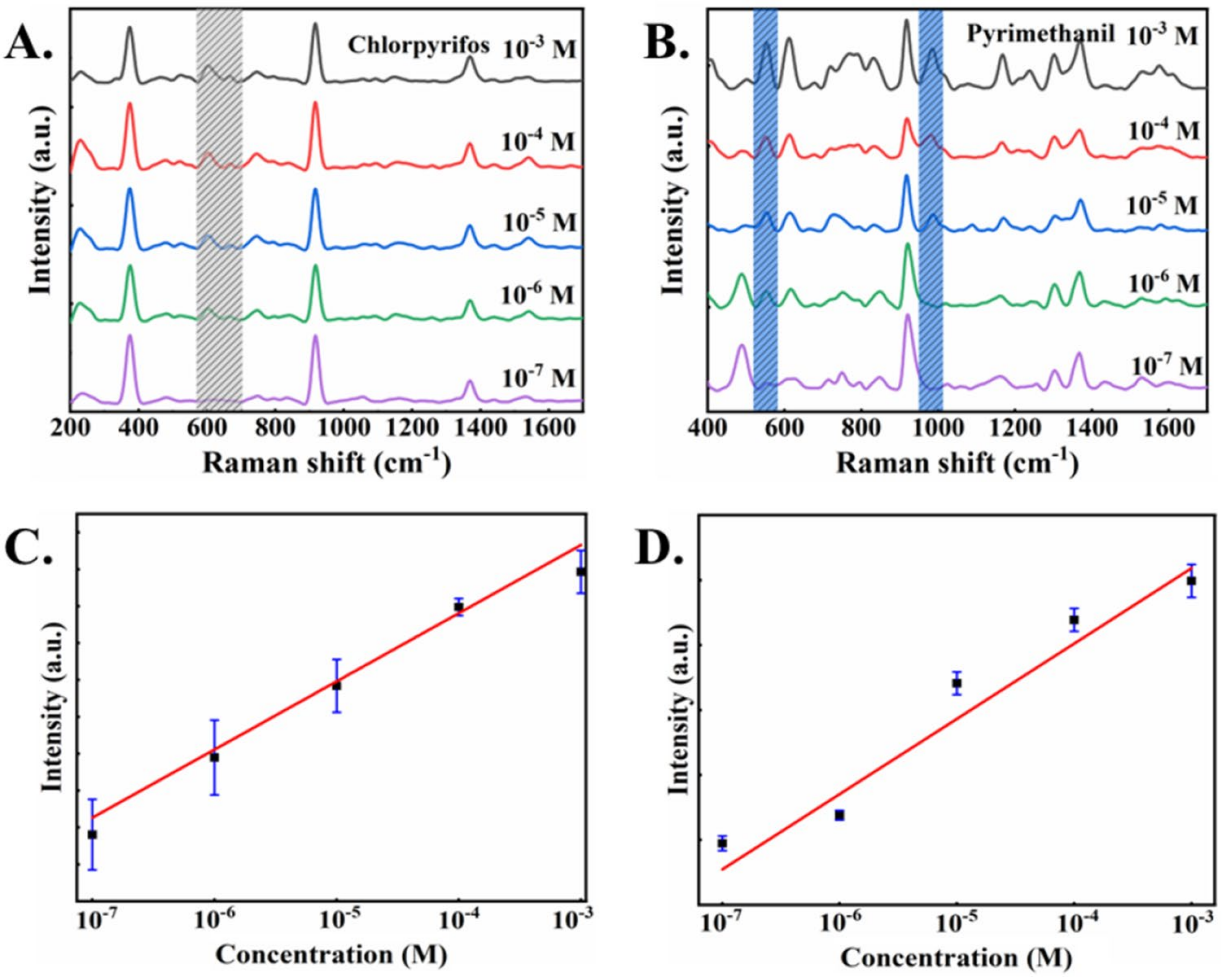
The SERS spectra of fruit surface treated with (**A**) chlorpyrifos and (**B**) pyrimethanil. (**C**) The relationship between the intensity of characteristic peaks at 609 cm^−1^ and the logarithm of concentration of chlorpyrifos in linear coordinates. (**D**) The relationship between the intensity of characteristic peaks at 558 cm^−1^ and the logarithm of concentration of pyrimethanil in linear coordinates.

The chlorpyrifos and pyrimethanil were mixed in different ratios (1:1, 1:2, 1:3, 1:4, 4:1, 3:1, 2:1) as for the preparation of the standard samples. Figure 4A,B show the SERS spectra of the tested chlorpyrifos and pyrimethanil standard solutions, respectively. For chlorphyrifos residue on the fruit surface, the SERS characteristic peaks at 609 cm^−1^ and 668 cm^−1^ could be clearly observed. Similarly, the SERS characteristic peaks of 559 cm^−1^, 990 cm^−1^ and 1294 cm^−1^ could be detected for pyrimethanil residue on the fruit surface. The lowest detection limits of chlorpyrifos and pyrimethanil residues on the fruit surface were estimated as 2.1 × 10^−4^ mg/cm^2^ and 1.2 × 10^4^ mg/cm^−2^, respectively, which is lower than the dangerous level of chlorpyrifos and pyrimethanil to human health (which is 1 mg kg^−1^ for chlorpyrifos and 7 mg kg^−1^ for pyrimethanil, respectively)^31,32^.

**Figure 5:**
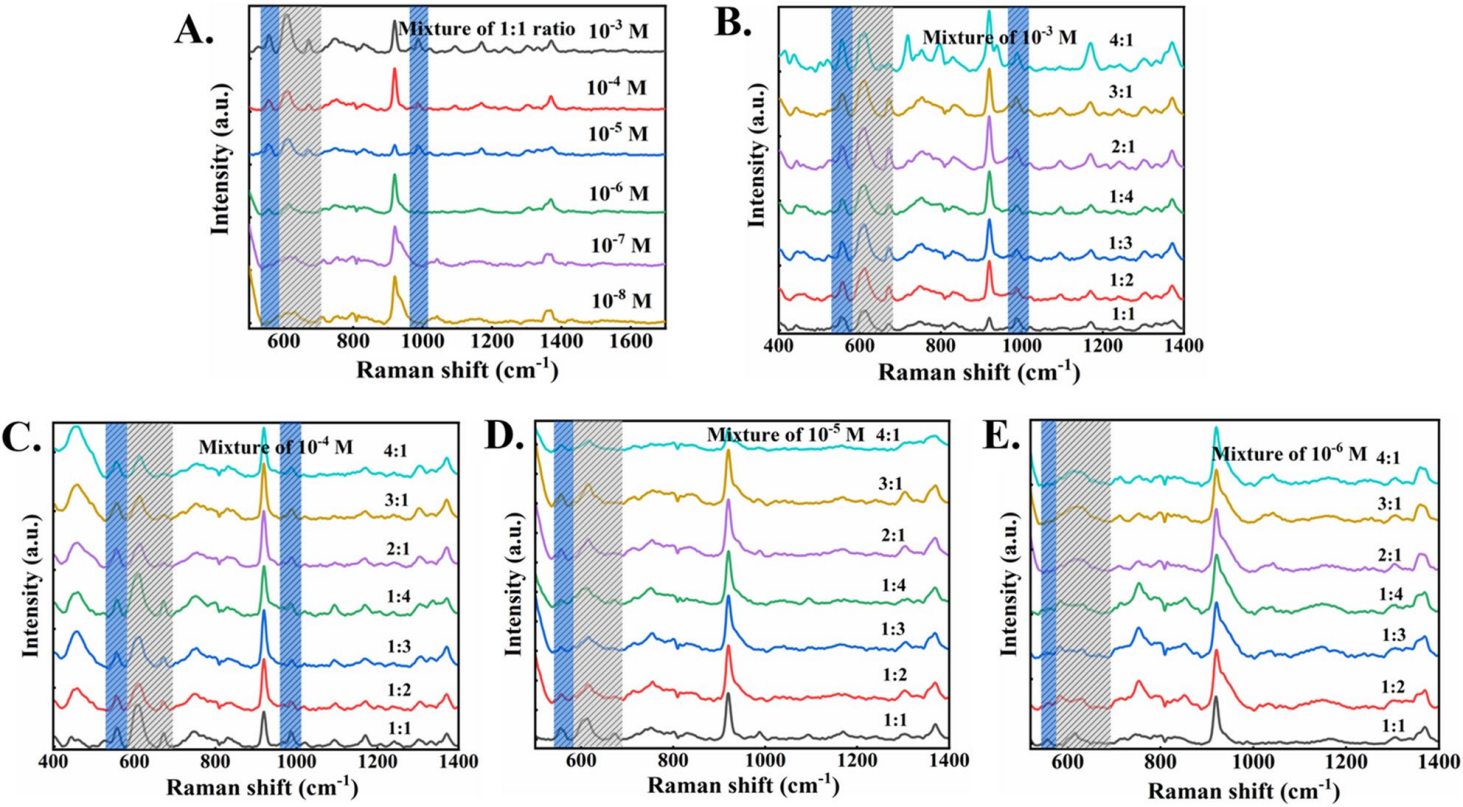
(**A**) The SERS spectra of fruit surface treated with equal volume mixed pyrimethanil/chlorpyrifos. (**B**) The SERS spectra of pyrimethanil/chlorpyrifos treated fruit surface with different volume ratios of 10^−3^ M, (**C**) 10^−4^ M, (**D**) 10^−5^ M, and (**E**) 10^−6^ M.

For the chlorpyrifos and pyrimethanil measurements, the SERS spectra show a certain positive correlation with the concentration of applied pesticide/fungicide. The SERS characteristic peaks at 609 cm^−1^ and 558 cm^−1^ were used to evaluate this relationship (Fig. 4C,D). It can be found that with the increase of pesticide/fungicide residue concentration on the fruits surface, the intensity of SERS characteristic peak also increases correspondingly. Figure 5A shows the SERS spectra for the mixture of pyrimethanil and chlorpyrifos (with equal volume), showing their characteristic bands clearly, indicating that the method can be used for simultaneous determination of mixed pesticide/fungicide residues on fruit surface.

Furthermore, Fig. 5B–E show the SERS spectra of the mixtures of pyrimethanil to chlorpyrifos with different mixing volume ratios. With the increasing ratio of pyrimethanil to chlorpyrifos, the intensity of characteristic peak of pyrimethanil at 558 cm^−1^ increased, while the characteristic peak intensity of chlorpyrifos at 675 cm^−1^ decreased. In the case of mixture measurements, the lowest detection limits of chlorpyrifos and pyrimethanil residues on fruit surface are 4.2 × 10^−4^ mg/cm^2^ and 2.4 × 10^4^ mg/cm^−2^, respectively. Similarly, for the mixed pesticide/fungicide residues  on the fruit surface, there is a certain relationship between SERS characteristic peak intensity and concentration. The overall results suggest that this SERS approach can detect mixed pesticide/fungicide residues on fruit surface with high sensitivity.

### Identification of mixed pesticide/fungicide residues

#### PCA analysis

**Figure 6:**
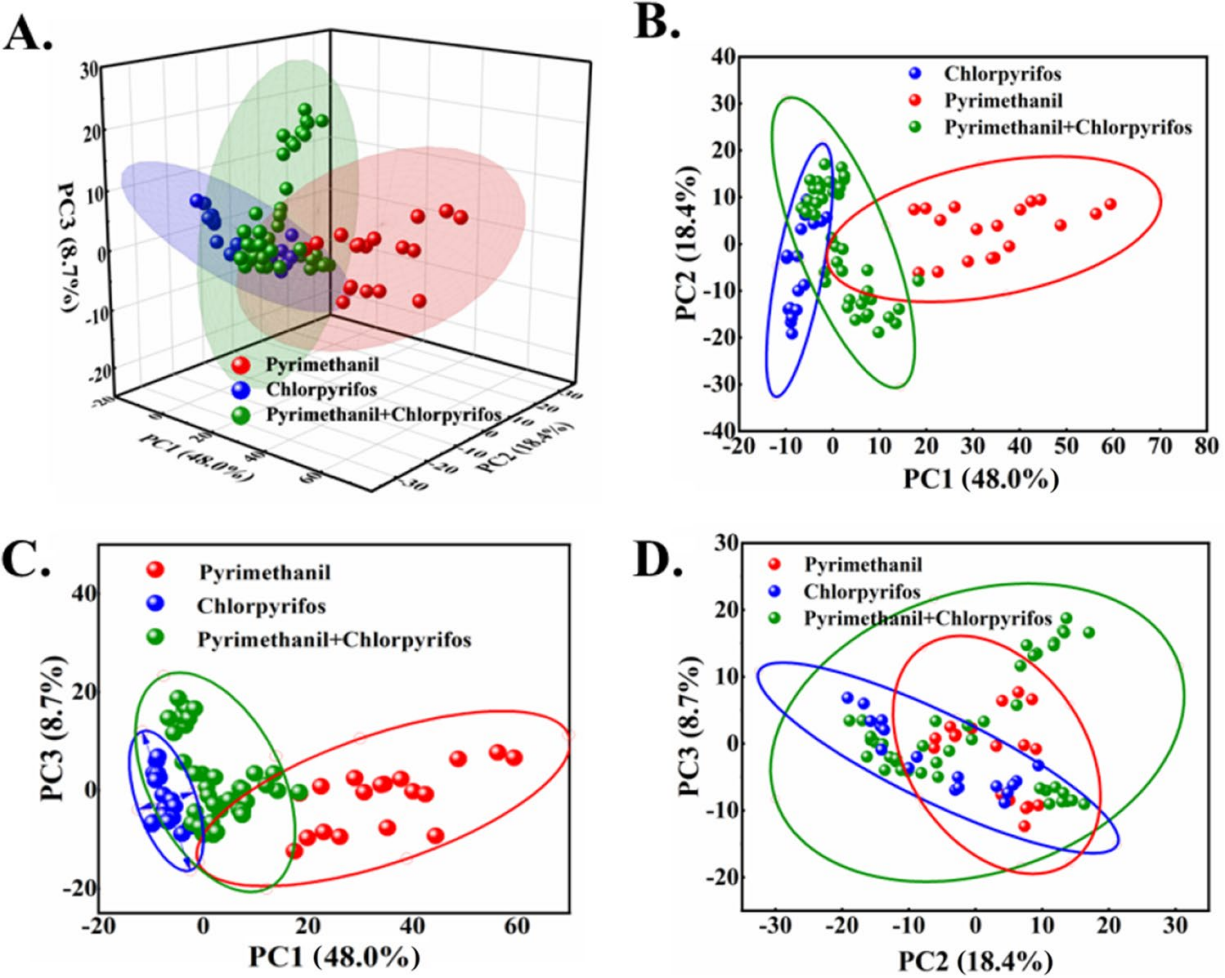
PCA results of SERS spectra of the samples treated with chlorpyrifos, pyrimethanil and mixture. (**A**) PC1-PC2-PC3 diagram. (**B**) PC1-PC2 diagram. (**C**) PC1-PC3 diagram. (**D**) PC2-PC3 diagram.

In order to explore the clustering of different pesticide/fungicide residues on fruits, principal component analysis (PCA) was conducted on the SERS spectral data of the tested solutions. The spectral ranges of 500–700 cm^−1^ and 950–1300 cm^−1^ are selected for the PCA analysis of the SERS spectra. The first three principal components that contribute the most to the variance are plotted and 95% confidence ellipses are added. The results are shown in Fig. 6.

According to the results of PCA, the variance contribution rate of PC1, PC2 and PC3 is 48.0%, 18.4% and 8.7%, respectively. The cumulative variance contribution rate of the three principal components (PC) can reach 75.1%, indicating that most of the data information can be retained in the coordinate space formed by them. According to the principal component results, the spectra of pyrimethanil and chlorpyrifos in PC1-PC2 and PC1-PC3 planes are clearly demarcated, and the aggregation among various categories is obvious. But the mixed residues are slightly overlapped. In the PC2-PC3 plane with the low contribution rate of cumulative variance, the three kinds of pesticide/fungicide residues overlap each other and there is no obvious separation boundary, indicating the limitation of distinguishing the mixed pesticide/fungicide residues by the PCA approach.

#### SVM classification model

**Figure 7:**
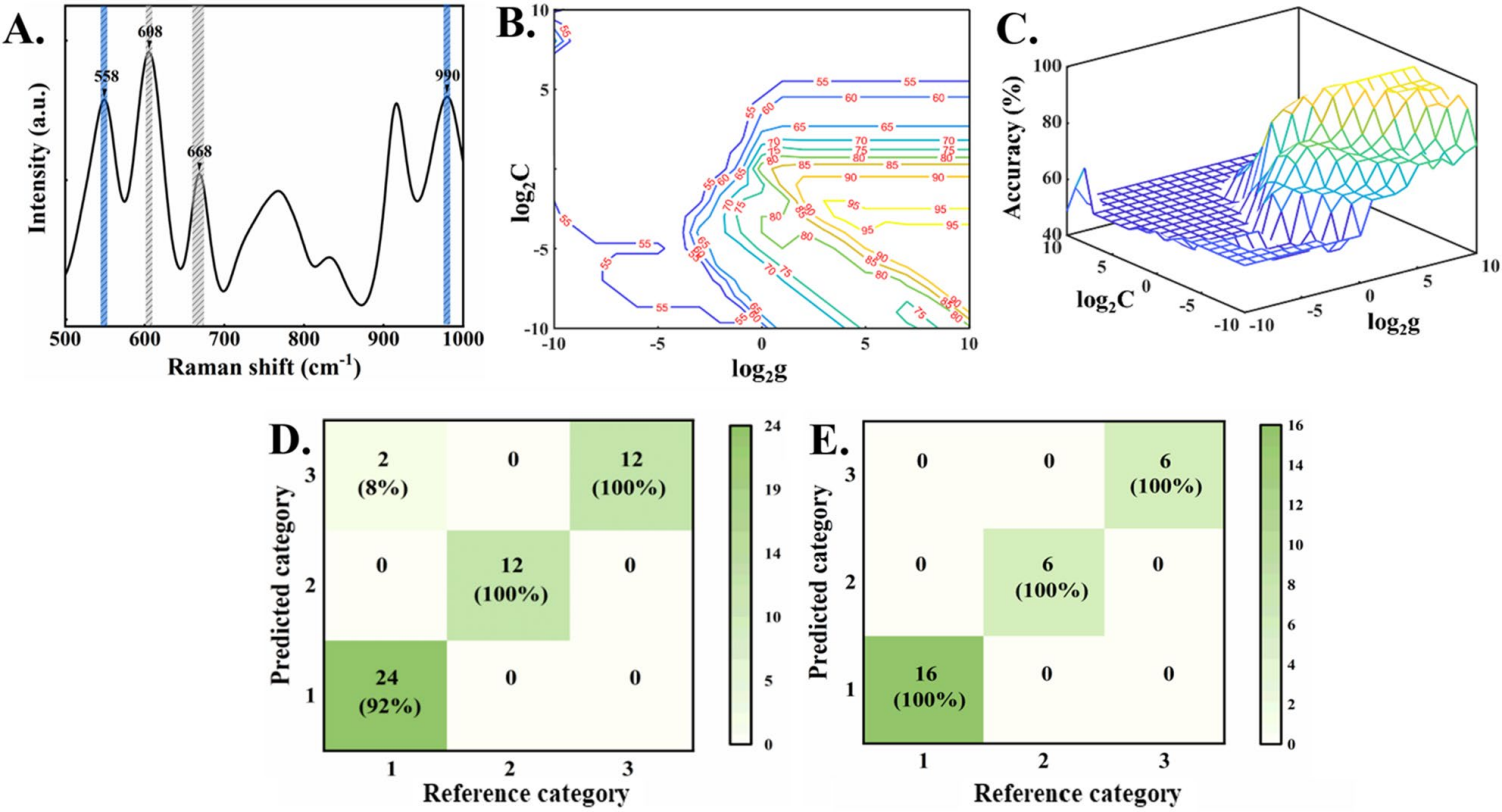
(**A**) The 55 wavenumber points selected in the spectrum. (**B**) Contour map of classification accuracy of SVM. (**C**) Hyperparameter C and g grid optimization. (**D**) Confusion matrix of the training set and (**E**) test set (Category 1 is mixed pesticide/fungicide residues, category 2 is pyrimethanil, category 3 is chlorpyrifos).

In order to solve the problem of inaccurate PCA classification, machine learning models were explored to extract nonlinear features in SERS spectral data. The SVM model can solve the problem of unclear classification of mixtures in limited-dimensional space by replacing the original nonlinear problem with the transformed linear problem through the nonlinear mapping of kernel function^33^. Due to the existence of a large number of irrelevant signals in the SERS spectra within the range of 200–1700 cm^−1^, the model might obtain too much noise information and ignore the target signal when the model is trained with full spectrum. Therefore, before establishment of SVM model, the characteristic peaks of chlorpyrifos and pyrimethanil (559 cm^−1^, 609 cm^−1^, 668 cm^−1^, 675 cm^−1^ and 990 cm^−1^) were selected as the center, and five wave points each side (55 wavenumbers) were used as the basic spectral dataset to build the model. The selected wavenumbers are shown in Fig. 7A.

In the assessment, the RBF (Radial Basis Function) kernel function was used to establish the nonlinear SVM classification model. It can deal with nonlinear problems that linear kernel function cannot deal with, and it has fewer parameters and wider application scenarios than the polynomial kernel function^34^. In the classification model of SVM, the kernel parameter g and penalty factor C are closely related to the classification boundary and performance of the model. In order to find the optimal parameters of C and g, accuracy is taken as the objective function and grid optimization is adopted to optimize the parameters of SVM model. The KS (Kennard-Stone) method is used to divide the samples into training sets and test sets in a 5:3 ratio according to previous work^35,36^. Among them, there are 50 training samples and 28 test samples, which are divided into chlorpyrifos, pyrimethanil and mixed pesticide/fungicide residues datasets for the model establishment. The key to improvement of the accuracy and generalization ability of the inversion model lies in the selection of kernel function parameter g and penalty factor C. Therefore, grid optimization method is adopted^37^. With classification accuracy as the objective function, penalty coefficient log_2_C and kernel function parameter log_2_g are optimized between -10 and 10. The optimization results are shown in Fig. 7B,C.

According to the contour line of classification accuracy, it can be found that the model with high classification accuracy is in the lower right corner of the data space composed. The larger g is, the more support vectors are and the more complex the model will be. Therefore, log_2_g and log_2_C with the highest accuracy and smaller parameters could be selected to establish the SVM classification model in the three-dimensional optimization space. Figure 7D,E show the confusion matrix of SVM model training set and test set. When the optimal parameter log_2_C is 4 and log_2_g is − 3, the accuracy of the training set is 96%, showing the good result in the detection and identification of chlorpyrifos and pyrimethanil residues in most cases, but it still has some errors for the mixtures with low concentrations.

#### CNN-GRU classification model

**Figure 8:**
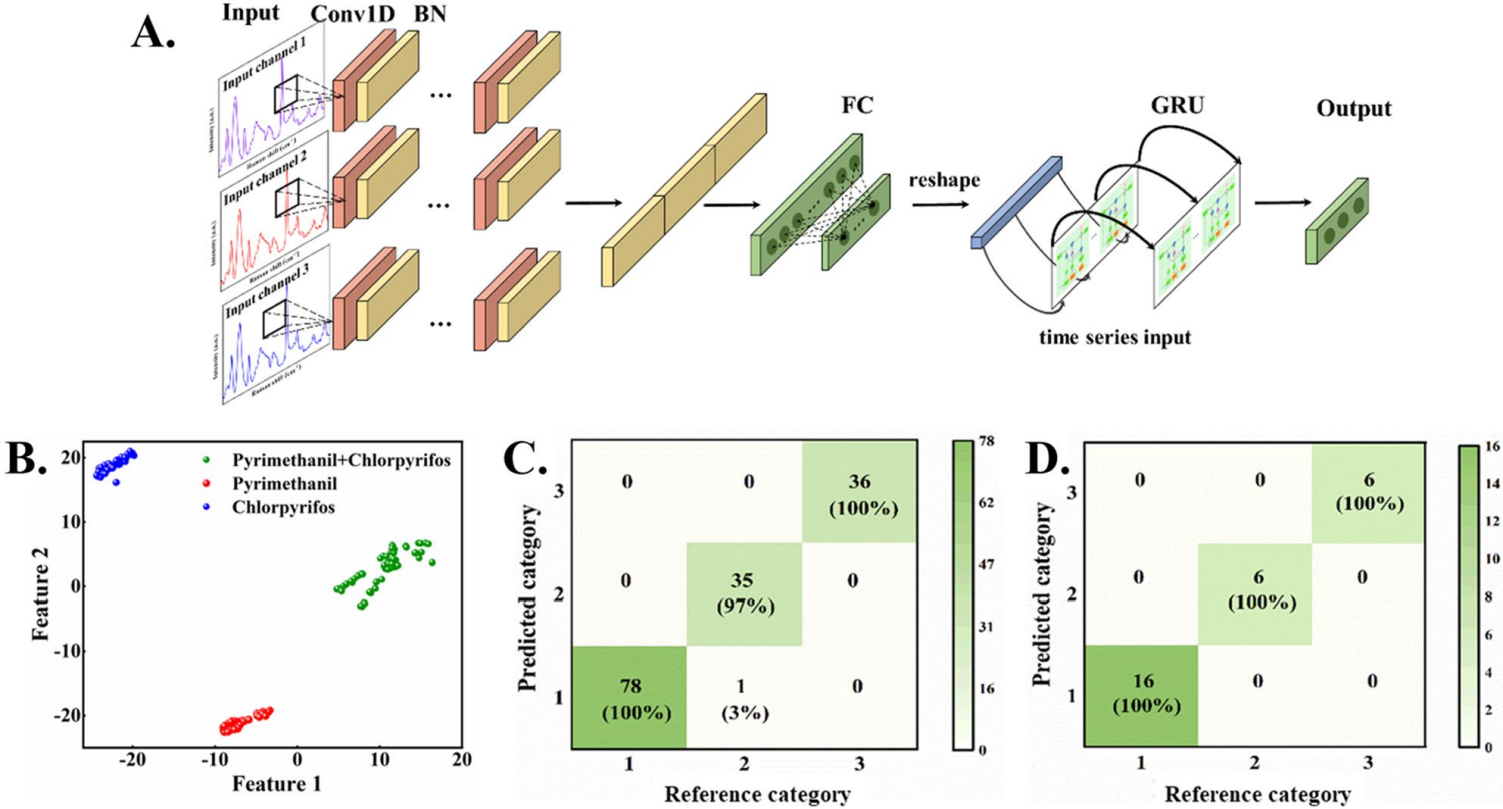
(**A**) MC-CNN-GRU discriminant model for pesticide/fungicide residues. (**B**) t-SNE analysis results of the MC-CNN-GRU model. (**C**) Confusion matrix of training set and (**D**) test set.

In order to further improve the prediction accuracy, MC-CNN-GRU was used as the exploration framework. After multi-channel input of spectral data, features could be extracted by the one-dimensional convolutional layer and GRU, so that the spatial information in the data could be collected. The spectra of multiple measurements of the same sample are slightly different. In order to eliminate the sample unevenness and improve the generalization performance of the model, the SERS spectra of the training set after SG smoothing, FFT filtering of the raw data are input into the one-dimensional CNN with the fixed kernel size through three channels, respectively. The test set is consistent with the SVM model. The input data of each channel are extracted by three one-dimensional convolution layers. To establish the model, 8 convolution layers, 16 and 32 convolution kernels were used to generate the corresponding number of feature vectors. The first two convolutional layers were followed by the batch normalization (BN) layer to avoid the disappearance of gradient, and integrated with the Swish function. The third convolutional layer was followed by the global pooling layer and the fully connected layer. Then the features were extracted by two GRU layers containing 16 and 32 hidden units. Finally, the discriminant types of pesticide/fungicide residues were obtained for the output. The structure is shown in Fig. 8A.

The multi-channel input was used to eliminate the errors caused by uneven samples, and the convolution and cycle units were used to extract spatial and sequence information. The Adam optimizer was used to train the model and the cross-entropy loss function was taken as objective function. The learning rate was updated iteratively and initial learning rate was set at 10^−3^. After 50 epoch training, the learning rate decreased to 1/10 of the initial rate. In addition, the model adopted the early stop mechanism, when the loss function of the model was less than 0.01, the model would stop training even if the training number was not reached, which thus could avoid the overfitting phenomenon of the model to a certain extent. As seen from the results, the MC-CNN-GRU model can better classify mixed pesticide/fungicide residues with higher accuracy compared with the SVM model in this case.

In order to further explore the performance of the model, the penultimate layer of the model was taken out and the t-SNE was used to visually cluster and visualize multidimensional features in two-dimensions on the premise of retaining the characteristics of high-dimensional features. The results are shown in Fig. 8B. Compared with the results of PCA, the boundary between the three categories of pesticide/fungicide residues is obvious and clear, showing the satisfactory result of qualitative analysis. Therefore, the mapping relationship between spectral data and category constructed by multiple feature extraction of Raman spectra, and the discriminant analysis of pesticide/fungicide residues is realized by using the nonlinear analysis ability of deep learning.

Therefore, as seen from Fig. 8C,D, the CNN-GRU model can effectively learn the feature information in the spectra, and the qualitative recognition accuracy in the training set and the test set can reaches 99% and 100%, respectively, achieving the classification task of pesticide/fungicide residues with high precision.

## Conclusions

In summary, this work demonstrated that mixed chlorpyrifos and pyrimethanil residues on the surface of fruit could be identified and analyzed via SERS combined with the deep learning model. The lower limit of detection of chlorpyrifos and pyrimethanil on the surface of fruit is 2 × 10^−4^ mg/cm^2^ and 1.2 × 10^−4^ mg/cm^2^, respectively. The MC-CNN-GRU model can extract the subtle characteristic information of SERS spectra, so that the recognition and quantification accuracy based on the optimized classification model could be greatly improved for the trace pesticide/fungicide residues at varied mixture ratios. Therefore, this work provides an effective approach to rapid detection and identification of different pesticides/fungicides coexisting in agricultural products and may be practically applied in fast food safety inspection tasks.

## Data Availability

The datasets generated during and/or analyzed during the current study are available from the corresponding author upon reasonable request.
